# Case Report: Optimizing Pre- and Intraoperative Planning With Hyperaccuracy Three-Dimensional Virtual Models for a Challenging Case of Robotic Partial Nephrectomy for Two Complex Renal Masses in a Horseshoe Kidney

**DOI:** 10.3389/fsurg.2021.665328

**Published:** 2021-05-31

**Authors:** Riccardo Campi, Francesco Sessa, Anna Rivetti, Alessio Pecoraro, Paolo Barzaghi, Simone Morselli, Paolo Polverino, Rossella Nicoletti, Vincenzo Li Marzi, Pietro Spatafora, Arcangelo Sebastianelli, Mauro Gacci, Graziano Vignolini, Sergio Serni

**Affiliations:** ^1^Unit of Urological Robotic Surgery and Renal Transplantation, Careggi Hospital, University of Florence, Florence, Italy; ^2^Department of Experimental and Clinical Medicine, University of Florence, Florence, Italy

**Keywords:** hyperaccuracy three-dimensional model, horseshoe kidney, planning, robotics, partial nephrectomy

## Abstract

**Objective:** To report a case of robot-assisted partial nephrectomy (RAPN) for two highly complex renal tumors in a patient with a Horseshoe kidney (HSK), focusing on the utility of hyperaccuracy three-dimensional (HA3D) virtual models for accurate preoperative and intraoperative planning of the procedure.

**Methods:** A 74-year-old Caucasian male patient was referred to our Unit for incidental detection of two complex renal masses in the left portion of a HSK. The 50 × 55 mm, larger, predominantly exophytic renal mass was located at the middle-lower pole of the left-sided kidney (PADUA score 9). The 16 × 17 mm, smaller, hilar renal mass was located at the middle-higher pole of the left-sided kidney (PADUA score 9). Contrast-enhanced CT scan images in DICOM format were processed using a dedicated software to achieve a HA3D virtual reconstructions. RAPN was performed by a highly experienced surgeon using the da Vinci Si robotic platform with a three-arm configuration. A selective delayed clamping strategy was adopted for resection of the larger renal mass while a clampless strategy was adopted for the smaller renal mass. An enucleative resection strategy was pursued for both tumors.

**Results:** The overall operative time was 150 min, with a warm ischemia time of 21 min. No intraoperative or postoperative complications were recorded. Final resection technique according to the SIB score was pure enucleation for both masses. At histopathological analysis, both renal masses were clear cell renal cell carcinoma (ccRCC) (stage pT1bNxMx and pT3aNxMx for the larger and smaller mass, respectively). At a follow-up of 7 months, there was no evidence of local or systemic recurrence.

**Conclusions:** Surgical management of complex renal masses in patients with HSKs is challenging and decision-making is highly nuanced. To optimize postoperative outcomes, proper surgical experience and careful preoperative planning are key. In this regard, 3D models can play a crucial role to refine patient counseling, surgical decision-making, and pre- and intraoperative planning during RAPN, tailoring surgical strategies and techniques according to the single patient's anatomy.

## Introduction

Horseshoe kidney (HSK) is the most frequent renal fusion anomaly, with an incidence of 0.15–0.25% in the general population; up to 12% of patients with HSK develop renal tumors, of which around 50% are renal cell carcinoma (RCC) (1, 2).

While surgical management of renal masses arising from HSKs can be highly challenging given their rarity and the lack of established guidelines (2), a recent multicenter study by the Young Academic Urologists (YAU) Renal Cancer working group showed that such tumors can be approached via both open and minimally invasive surgery, with maximal preservation of functional renal parenchyma and acceptable histopathological and perioperative outcomes (1).

This surgery being highly demanding, meticulous pre-surgical planning and taking advantage of advanced imaging techniques and three-dimensional (3D) models (3, 4) have been advocated to aid in achieving good outcomes (1, 5). Specific challenges associated with nephron-sparing approaches for tumors arising from HSKs are represented by the limited possibility to mobilize the kidney, the difficulty in recognizing and controlling the several vascular structures of the renal hilum (which are highly variable across patients), and often the need for complex renal reconstruction techniques (6).

As such, optimizing pre- and intra-operative planning by means of 3D models (reproducing the patient-specific renal and vascular anatomy) to allow surgeons to pursue nephron-sparing techniques in case of highly complex renal masses arising from HSKs is an unmet need.

Herein, we report a case of robot-assisted partial nephrectomy (RAPN) for two complex renal tumors in a patient with a HSK, focusing on the utility of 3D virtual models for accurate preoperative and intraoperative planning of the procedure.

## Case Report

### Case Presentation

A 74-year-old Caucasian male patient was referred to our Unit for incidental detection of two complex renal masses in the left portion of a HSK at computed tomography (CT) imaging performed for follow-up of a previously treated laryngeal carcinoma (clinical remission for 4 years after surgery plus adjuvant radiotherapy). The presence of a HSK was not known.

No local or systemic symptoms that could be related to the renal masses were present.

The patient's surgical history included also a cardiosurgical intervention for aortic valve replacement, currently requiring antiplatelet therapy, and left inguinal hernia repair. Patient's comorbidities included hypertension and mild dyslipidemia (both controlled with medical therapy). The patient's age-adjusted Charlson Comorbidity Index was 3.

At physical examination, no palpable flank masses could be detected. Body mass index was 22 kg/m^2^. Preoperative renal function was preserved [estimated glomerular filtration rate (eGFR): 74 ml/min/m^2^], and all biochemical parameters were within normal ranges. No hematuria or proteinuria were recorded.

### Tumor Nephrometric Characterization

**Figure 1:**
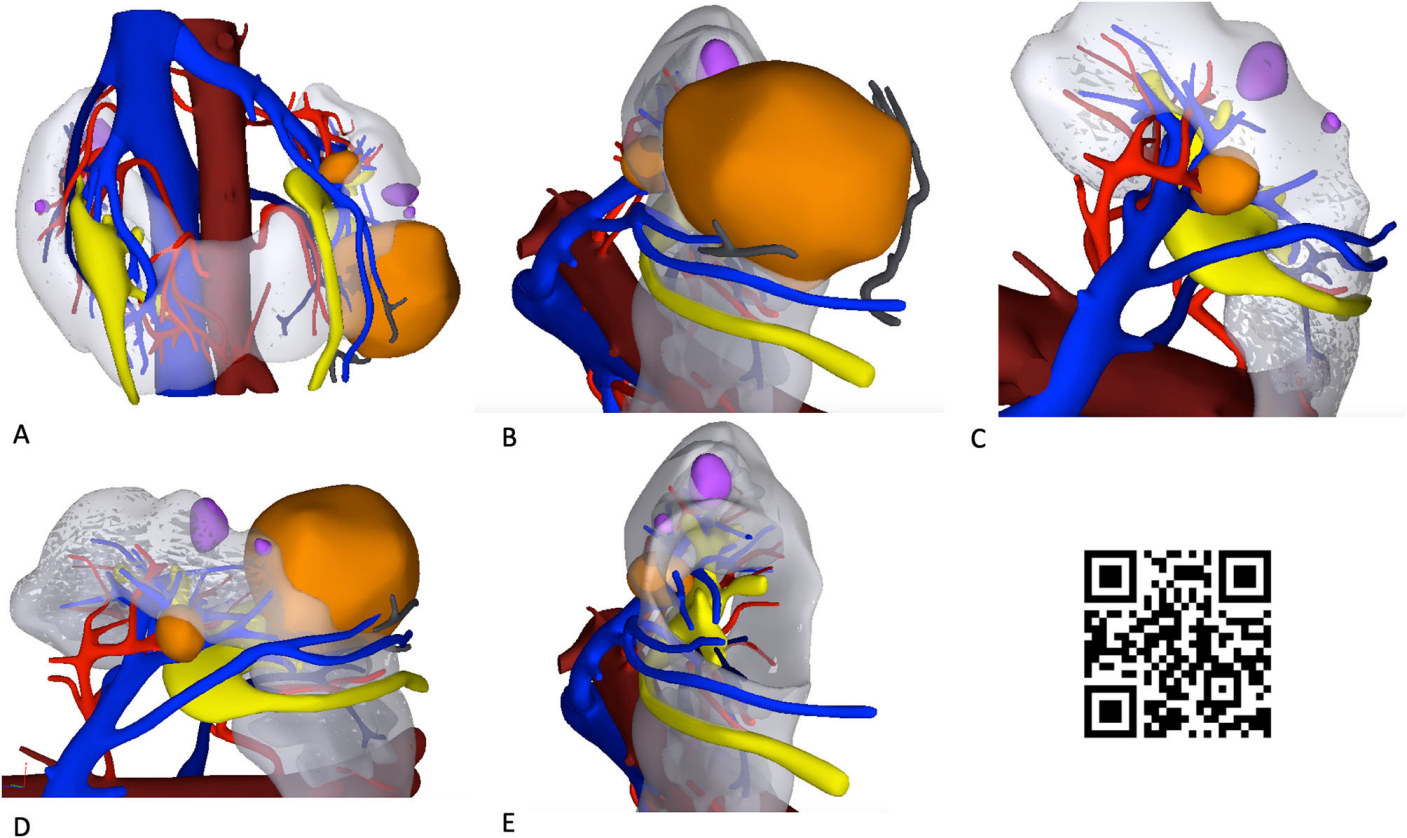
Hyperaccuracy three-dimensional (HA3D) virtual model of the two renal masses in a patient with a horseshoe kidney (HSK). The snapshots **(A–E)** show the anatomical relationships of the two renal masses (colored orange) with the extra- and intra-renal vascular (veins, in blue; arteries, in red) and urinary structures (in yellow). Simple renal cysts are colored violet. A linked video clip is available scanning the QR code on the right side of the image. For apple users: open the Camera app from your devices. Hold your device so that the QR code appears in the Camera app's viewfinder. Your device recognizes the QR code and shows a notification. Tap the notification to open the link associated with the QR code. For Android users: download a QR code scanner app and follow the above instructions.

Abdominal contrast-enhanced CT scan revealed two left-sided renal masses in the HSK (Figure 1).

The 50 × 55 mm, larger, predominantly exophytic renal mass was located at the middle-lower pole of the left-sided kidney. Tumor complexity was classified as intermediate (PADUA score of 9: 2 points for “tumor size”; 1 point for “exophytic” rate; 2 points for “collecting system” involvement; 1 point for “sinus” involvement; 1 point for “renal rim”; 2 points for polar location).

The 16 × 17 mm, smaller, hilar renal mass was located at the middle-higher pole of the left-sided kidney, and was in contact with arterial branches and the renal sinus. Tumor complexity was also judged as intermediate (PADUA score 9: 1 point for “tumor size”; 1 point for “exophytic” rate; 1 point for “collecting system” involvement; 2 points for “sinus” involvement; 2 points for “renal rim”; 2 points for polar location). Clinical stage was cT1b N0M0 and cT1a N0M0, respectively.

### Hyperaccuracy Three-Dimensional (HA3D) Virtual Model

Contrast-enhanced CT scan images in DICOM format were processed by MEDICS Srl (www.medics3d.com) using dedicated software to achieve a HA3D virtual reconstruction of the case, as previously reported (4, 7).

Specifically, the process included the creation of a 3D virtual model rendering of the HSK based on high-resolution CT scan images. After reconstruction of the anatomy of the HSK and of the two renal masses, careful evaluation of the renal vasculature and urinary collecting system was performed (Figure 1).

The virtual navigation of the HA3D model allowed the surgeon to appreciate the anatomical details of the two complex renal masses, focusing on their relationships with the vascular arterial and venous branches arising from the aorta and the inferior vena cava, as well as with the intrarenal portion of the urinary collecting system. Of note, navigating the 3D-pdf file after HA3D model rendering fostered a careful evaluation of the preoperative surgical strategy by the whole surgical team (Data Sheet 1).

### Patient Counseling and Preoperative Planning

The clinical case was discussed by our institutional multidisciplinary tumor board. After careful discussion of the available options (i.e., percutaneous CT-guided renal tumor biopsy of the largest mass followed by surgery or ablation in case of a malignant tumor; surgery with curative intent without previous renal tumor biopsy; percutaneous ablation of the largest renal mass and active surveillance of the smaller renal mass), the patient was offered surgery with curative intent without preoperative tumor biopsy. During preoperative counseling, the potential benefits and harms of both partial and radical nephrectomy were discussed with the patient, taking into account his values and preferences. Regardless of the type of nephrectomy, the patient was offered a minimally invasive approach (robotic surgery). The patient finally opted for RAPN for both renal masses.

A written informed consent was collected before writing this manuscript.

**Figure 2:**
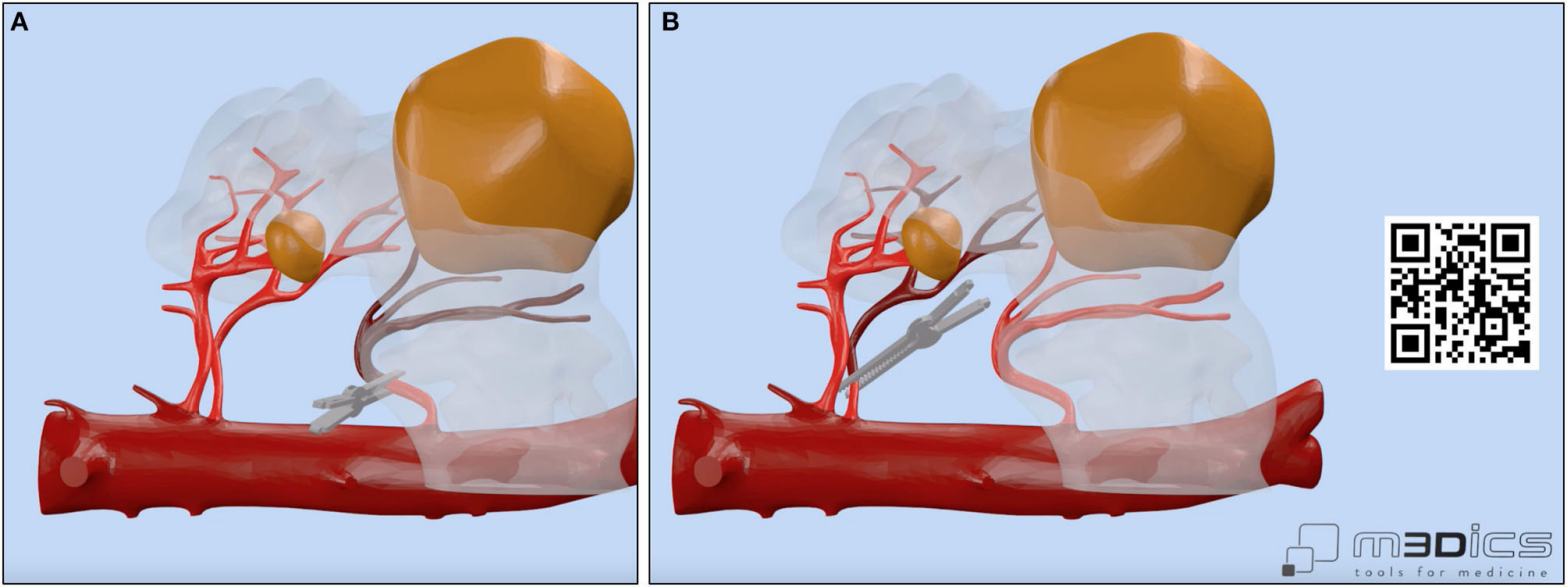
Vascular anatomy of our case with focus on the main renal arteries feeding the left-sided portion of the horseshoe kidney (HSK) and on the clamping strategy adopted by the surgeon. A selective delayed clamping strategy was performed for resection of the larger renal mass **(A,B)** while a clampless strategy was chosen for resection of the smaller renal mass. A linked video clip is available scanning the QR code on the right side of the image. For Apple users: open the Camera app from your devices. Hold your device so that the QR code appears in the Camera app's viewfinder. Your device recognizes the QR code and shows a notification. Tap the notification to open the link associated with the QR code. For Android users: download a QR code scanner app and follow the above instructions.

Our preoperative surgical strategy involved the following points:

(a) use of the da Vinci Si robotic platform with a three-arm configurations plus two assistant ports [both 12-mm ports, of which one is for the AirSeal® system (SurgiQuest, ConMed Corporation, Milford, CT)] (in this specific case, we employed two 12-mm ports for the assistant to allow him to use vascular clamps and hem-o-lok clips from both ports, maximizing patient safety and coordination with the primary surgeon);
(b) transperitoneal approach;
(c) selective delayed clamping for resection of the larger renal mass (Figure 2) and clampless resection of the smaller renal mass;
(d) enucleative resection strategy (8, 9), aiming to achieve tumor enucleation according to the Surface-Intermediate-Base (SIB) margin score (10);
(e) double-layer and single-layer renorrhaphy for renal reconstruction of the larger and smaller renal mass, respectively (6, 11).

Notably, the opportunity to navigate the HA3D virtual model (Figure 1) allowed the surgeon to take advantage of the knowledge of the anatomical relationships of the tumor with the vascular and urinary systems to tailor the surgical strategy for tumor resection both preoperatively and intraoperatively. In particular, the 3D model allowed us to appreciate the anatomy of the three renal arteries originating from the aorta and feeding the left kidney, orienting the surgeon toward a selective clamping strategy for resection of the larger mass and a clampless strategy for resection of the smaller mass (Figure 2).

### Surgical Technique for RAPN

**Figure 3:**
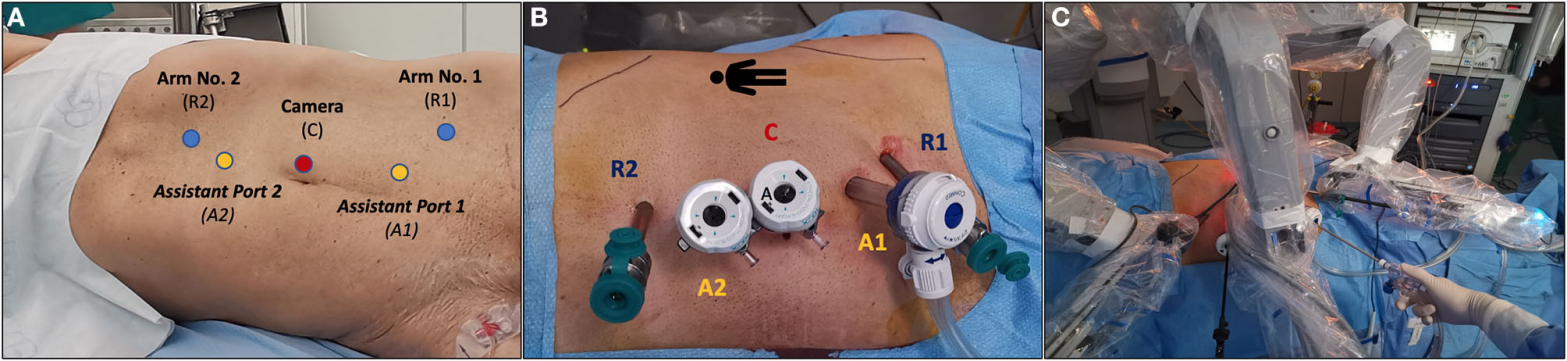
Overview of port placement in our case of robot-assisted partial nephrectomy (RAPN) for two complex renal masses in a patient with a horseshoe kidney. The da Vinci Si robotic platform with a 30° lens, a three-arm configuration plus two assistant ports [both 12-mm ports, of which one for the AirSeal® system (SurgiQuest, ConMed Corporation, Milford, CT)] was adopted. **(A)** Placement of the camera port using the Hasson technique plus two robotic ports and two 12-mm assistant ports. **(B)** Overview of the final port placement. **(C)** Docking of the robot.

RAPN was performed by a highly experienced robotic surgeon (S.S., >1,500 robotic urological procedures and >500 PNs) using the da Vinci Si platform (Intuitive Surgical, Sunnyvale, CA) with a 30° lens in a three-arm configuration. Port placement was performed following established principles (12), with a few technical modifications allowing to optimize the access to the HSK and to mobilize the left kidney properly (Figure 3). Pneumoperitoneum was set at 12 mmHg and maintaining constant during the procedure thanks to the Air Seal system.

**Figure 4:**
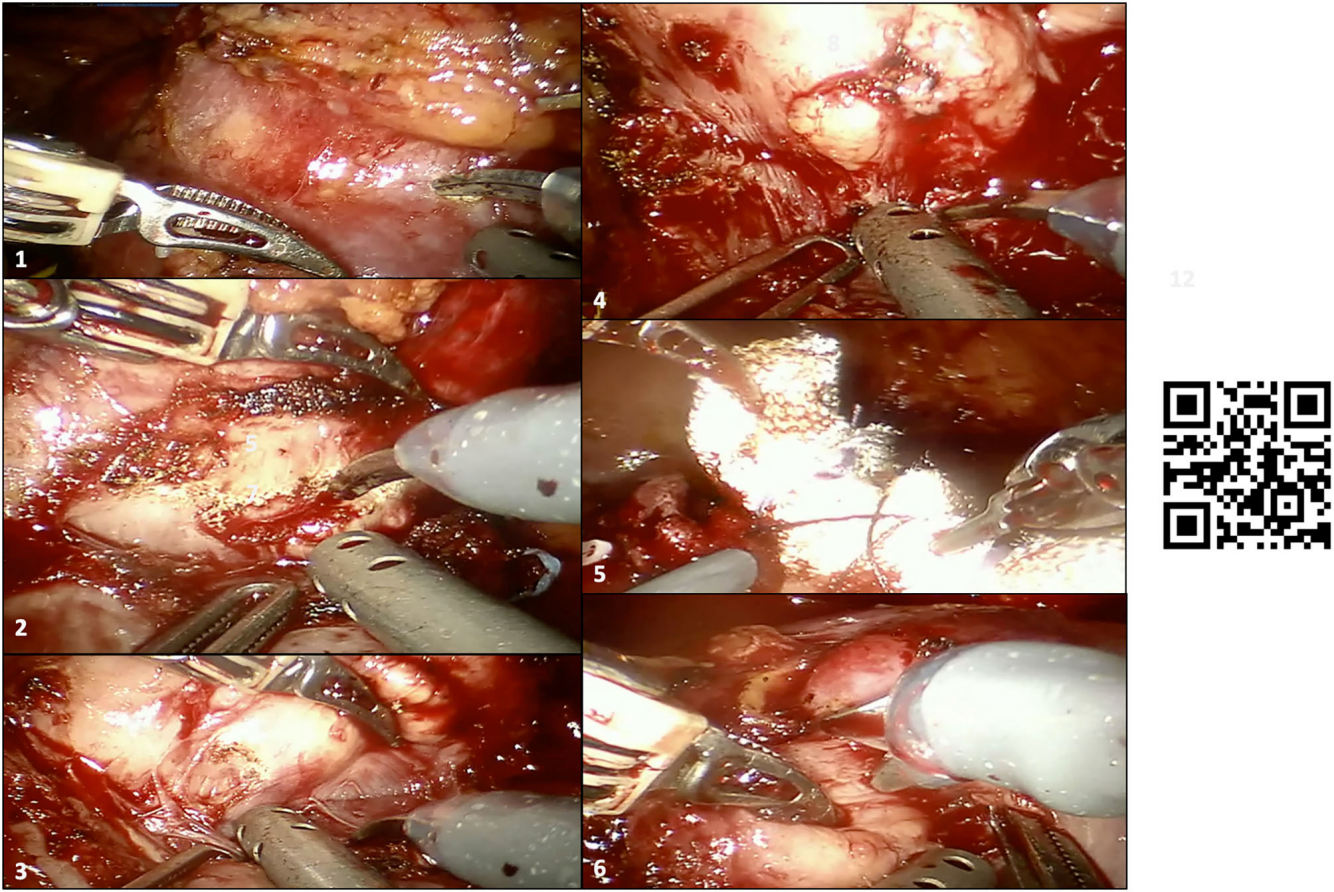
Intraoperative snapshot showing the main steps of robot-assisted partial nephrectomy (RAPN) in our case. (1–4) Development of the enucleation plane for resection of the larger renal mass (before clamping the renal hilum), with inadvertent capsulotomy of the tumor during tumor enucleation after clamping of the renal hilum (2); the surgeons recognized the wrong plane of dissection and continued the enucleation through the real anatomic plane between the tumor and the healthy renal parenchyma with no macroscopic positive margins; (5) double-layer renorrhaphy to close the renal defect after tumor enucleation of the larger renal mass; (6) enucleation of the smaller hilar renal mass, which was in contact with both vascular structures and the sinus fat. A linked video clip is available scanning the QR code on the right side of the image. For Apple users: open the Camera app from your devices. Hold your device so that the QR code appears in the Camera app's viewfinder. Your device recognizes the QR code and shows a notification. Tap the notification to open the link associated with the QR code. For Android users: download a QR code scanner app and follow the above instructions.

Our technique for RAPN has been previously described (12), while a detailed step-by-step overview of RAPN with HA3D model-guided planning in our case is shown in Figure 4.

Briefly, after medialization of the left colon and identification of the left kidney, all three renal arteries feeding the left kidney were carefully identified and dissected. Then, after controlling for the left gonadal vessels and several small accessory vessels directed to the larger renal mass, the lower and middle portions of the left kidney were freed from the surrounding adipose tissue and the larger renal mass was accurately isolated. Of note, the HA3D virtual model was always available for the operating surgeon to refine the intraoperative decision-making, to better recognize anatomical structures, and to guide the planning of the main steps of RAPN. The larger renal mass was approached first. After delineation of its contours, and once the “enucleation” plane has been identified, the two renal arteries feeding the tumor-bearing portion of the left kidney were clamped (Figure 2) and tumor resection was carried out using an enucleative resection strategy, as previously described (9) (Figure 4).

In particular, once a radial nephrotomy 1 to 2 mm from the lesion has been made, the natural, relatively avascular anatomic dissection plane between the peritumoral pseudocapsule and healthy renal parenchyma was developed by blunt dissection using circumferential, dynamic tractions with the two robotic arms that lifted the tumor off the parenchymal bed (12).

Once the tumor was enucleated, a double-layer renorrhaphy was achieved following established technical principles (12), aiming to maximize the quantity of vascularized parenchyma preserved (6, 11). Then, the two renal arteries were unclamped, and robotic tumor enucleation of the smaller renal mass was performed using a clampless anatomic resection strategy, preserving all vascular branches feeding the middle-upper pole of the left kidney. Hemostasis was achieved with a single-layer renorrhaphy and the Floseal® (Baxter Healthcare Corporation Fremont, CA) hemostatic matrix.

### Intra- and Post-operative Outcomes

The overall operative time for RAPN in our case was 150 min, with a warm ischemia time (WIT) of 21 min. The estimated blood loss was 160 ml. No intraoperative complications occurred.

The resection technique for both renal masses was classified as pure enucleation (SIB score 0 for the larger mass and SIB score 1 for the smaller mass).

The patient underwent a pre-planned postoperative stay in the Intensive Care Unit for 24 h, after which he was re-admitted in our Urology Unit. The postoperative course was uneventful, with the urinary catheter removed on postoperative day 1 and the surgical drain on postoperative day 2.

The patient was discharged from the hospital on postoperative day 4 in good clinical conditions and with an eGFR of 66 ml/min/1.73 m^2^.

### Histopathological Analysis

Handling of the specimen and histopathological analysis were performed by dedicated uropathologists according to standardized Institution protocols (12).

Tumor stage was classified according to the 2010 TNM criteria; nucleolar grading and histopathological classification of RCC followed the most recent International Society of Urological Pathology recommendations (13, 14). Positive surgical margins were defined as presence of neoplastic cells directly in contact with the inked surface of the specimen.

At histopathological analysis, the larger renal mass was revealed to be a 52 × 50 mm, G3, clear cell renal cell carcinoma (ccRCC) with evidence of necrosis and negative surgical margins (pT1bNxMx), while the smaller mass was a 20 × 13 mm, G3 ccRCC without necrosis with a focal involvement of the sinus fat and negative surgical margins (pT3aNxMx) (Supplementary Figure 1).

### Follow-Up

Follow-up after RAPN was scheduled according to established guidelines (2).

At a follow-up of 7 months, the patient was free of symptoms and did not experience any postoperative surgical or medical complication. At the first imaging evaluation with contrast-enhanced CT scan 6 months after surgery, there were no signs of local or systemic recurrence.

## Discussion

Current guidelines recommend partial nephrectomy for the treatment of T1 renal masses in patients who are candidates for surgery and for whom a nephron-sparing approach is deemed technically and oncologically feasible (2). In this scenario, RAPN is being increasingly performed in high-volume referral centers worldwide (15).

Notably, renal tumors arising from HSKs represent a challenge due to their rarity and complex surgical and vascular anatomy (1); such cases mandate a careful preoperative planning to optimize surgical strategy and postoperative outcomes.

In this regard, the use of 3D models for preoperative and intraoperative planning of RAPN in case of complex renal masses has been shown to have an impact on postoperative outcomes (4, 5, 7, 16). As such, 3D models are increasingly used for planning of complex RAPN in routine surgical practice (16), and pioneering studies from selected referral institutions demonstrated the feasibility of using 3D augmented-reality guidance during RAPN to tailor tumor resection and renal reconstruction according to the specific anatomy of each patient, aiming to obtain “precision RAPN” and to improve postoperative functional outcomes (7, 17).

In this manuscript, we reported a case of RAPN for two complex renal masses in a patient with a HSK, focusing on the utility of HA3D virtual models for accurate preoperative planning and intraoperative guidance to optimize the precision of tumor excision and renal reconstruction, aiming to optimize the quantity of vascularized parenchyma preserved during partial nephrectomy.

While being a case report, our experience underlines the distinct benefits of integrating 3D models in preoperative and intraoperative planning of demanding RAPNs for challenging renal masses, such as those arising from HSKs (Figures 1, 2, 4).

A first key advantage of incorporating patient-specific HA3D models in the surgeon's strategy is to overcome the limitations associated with the (two-dimensional) interpretation of conventional contrast-enhanced CT images, allowing surgeons to readily focus on the key anatomical elements of the patient's tumor and kidney, including the extra- and intrarenal vascularization [which is often highly complex in HSKs (1)]. By navigating the 3D virtual model (Figure 1), even highly experienced surgeons can plan the main steps of RAPN, such as the clamping strategy (Figure 2) tumor excision (8) and renal reconstruction (6) (including potential pelvicalyceal repair), more effectively. Of note, the potential role of 3D models to modulate the clamping strategy might be of great value for surgeons, given the current controversies regarding the ultimate factors driving the decision to clamp or not to clamp during RAPN even in experienced hands (18). In our case, the navigation of the HA3D model allowed us to understand the details of renal vascular and pelvicalyceal anatomy, guiding a selective clamping strategy to reduce the WIT and precise renal reconstruction after tumor enucleation (Figure 2). Our experience is in line with the results of previous studies showing that the use of virtual 3D models in the operating rooms did not distract from the procedure and provided useful additional information for surgery guidance purposes (19).

Second, the use of HA3D virtual models allows us to easily discuss and communicate the surgical strategy between the members of the surgical team (operating surgeon, assistant, scrub nurse, etc.), with critical implications for intraoperative teamwork. As a corollary, integration of 3D models in the preoperative planning of complex RAPN allows us to successfully teach residents and trainees the main steps of the procedure, allowing them to appreciate the complexity of decision-making and the rational for each surgical step. This “educational” value of 3D virtual models was not limited to surgeons and trainees, but was also highly appreciated by the patient during preoperative counseling. A patient-specific anatomical 3D model might therefore facilitate a transparent communication of the potential benefits and harms of each available therapeutic options (i.e., partial vs. radical nephrectomy) in the single patient setting (20).

A third key advantage of using 3D models for preoperative planning of complex RAPNs, especially in challenging scenarios such as HSKs, is represented by the ability to foster a “virtual” surgery before actually performing it in the operating room. This allows one not only to refine the decision-making on resection and reconstruction techniques but also to “anticipate” potential intraoperative challenges that may require rapid and effective interventions (i.e., identification of aberrant renal vessels, clamping of selective renal artery branches, better control of the renal hilum in case of bleeding, etc.). Of note, previous studies have shown that the use of 3D augmented-reality guidance during RAPN led to higher rates of tumor enucleation and clampless interventions, as well as higher renal function preservation, as compared to “conventional” RAPN with intraoperative ultrasound guidance (7). Given the benefits of tumor enucleation as compared to other resection techniques (6, 8), the potential influence of 3D models on surgical decision-making regarding the resection strategy during complex RAPN warrants further investigations.

In conclusion, the surgical management of complex renal masses in patients with HSKs is challenging and decision-making is highly nuanced given the rarity of these tumors and the lack of established guidelines. While the current evidence is limited, recent reports highlight the feasibility and safety of minimally invasive approaches to perform nephron-sparing surgery in such demanding scenarios (1, 21, 22). To optimize postoperative outcomes, proper surgical experience and careful preoperative planning are key. In this regard, despite its limitations (single-case study employing a technology that has already been used in kidney cancer surgery), our report outlines that 3D models can play a crucial role to refine patient counseling, surgical decision-making, and pre- and intraoperative planning during RAPN, tailoring surgical strategies and techniques according to the single patient's anatomy. To this aim, our report confirms the feasibility and safety of RAPN for complex scenarios such as tumors arising from HSKs provided proper surgeon's and center's experience (23). Further research is needed to evaluate the added value of 3D models for preoperative planning of “precision” RAPN and their ultimate impact on surgical complications (24) and perioperative functional outcomes (25), taking into account surgeon experience, center volume, and renal mass complexity (26).

## Data Availability Statement

The original contributions generated for the study are included in the article Supplementary Material, further inquiries can be directed to the corresponding author/s.

## Ethics Statement

Ethical review and approval was not required for the study on human participants in accordance with the local legislation and institutional requirements. The patients/participants provided their written informed consent to participate in this study. Written informed consent was obtained from the individual(s) for the publication of any potentially identifiable images or data included in this article.

## Author Contributions

RC, FS, and SS: study design. AR, PB, AP, PP, and SM: video editing. AR, PB, and AP: data collection. RC, FS, and AR: manuscript writing. MG, VL, GV, AS, PS, and RN: critical revision of the manuscript. SS: supervision. All authors contributed to the article and approved the submitted version.

## Conflict of Interest

The authors declare that the research was conducted in the absence of any commercial or financial relationships that could be construed as a potential conflict of interest.

## Supplementary Material

Supplementary Figure 1 Overview of the two renal masses resected in our case at both contrast-enhanced computed tomography (CT) scan **(A-C)** and histopathological analysis **(D-G)**. The larger renal mass was revealed to be a 52 x 50 mm, G3, clear cell renal cell carcinoma (ccRCC) with evidence of necrosis and negative surgical margins (pT1bNxMx) (despite the presence of a small capsulotomy – see Figure 4) while the smaller mass a 20 x 13 mm, G3 ccRCC without necrosis with a focal involvement of the perirenal fat and negative surgical margins (pT3aNxMx).

## References

[1] Roussel E Tasso G Campi R Kriegmair MC Kara Ö Klatte T. Surgical management and outcomes of renal tumors arising from horseshoe kidneys: results from an international multicenter collaboration. Eur Urol. (2021) 79:133–40. 10.1016/j.eururo.2020.09.012 32950296

[2] Ljungberg B Albiges L Abu-Ghanem Y Bensalah K Dabestani S Fernández-Pello S. European association of urology guidelines on renal cell carcinoma: the 2019 update. Eur Urol. (2019) 75:799–810. 10.1016/j.eururo.2019.02.011 30803729

[3] Cacciamani GE Shakir A Tafuri A Gill K Han J Ahmadi N. Best practices in near-infrared fluorescence imaging with indocyanine green (NIRF/ICG)-guided robotic urologic surgery: a systematic review-based expert consensus. World J Urol. (2020) 38:883–96. 10.1007/s00345-019-02870-z 31286194

[4] Porpiglia F Fiori C Checcucci E Amparore D Bertolo R. Hyperaccuracy three-dimensional reconstruction is able to maximize the efficacy of selective clamping during robot-assisted partial nephrectomy for complex renal masses. Eur Urol. (2018) 74:651–60. 10.1016/j.eururo.2017.12.027 29317081

[5] Shirk JD Thiel DD Wallen EM Linehan JM White WM Badani KK. Effect of 3-dimensional virtual reality models for surgical planning of robotic-assisted partial nephrectomy on surgical outcomes: a randomized clinical trial. JAMA Netw Open. (2019) 2:e1911598. 10.1001/jamanetworkopen.2019.11598 31532520 PMC6751754

[6] Bertolo R Campi R Klatte T Kriegmair MC Mir MC Ouzaid I. Suture techniques during laparoscopic and robot-assisted partial nephrectomy: a systematic review and quantitative synthesis of peri-operative outcomes. BJU Int. (2019) 123:923–46. 10.1111/bju.14537 30216617

[7] Porpiglia F Checcucci E Amparore D Piramide F Volpi G Granato S. Three-dimensional augmented reality robot-assisted partial nephrectomy in case of complex tumours (PADUA ≥10): a new intraoperative tool overcoming the ultrasound guidance. Eur Urol. (2020) 78:229–38. 10.1016/j.eururo.2019.11.024 31898992

[8] Minervini A Campi R Lane BR De Cobelli O Sanguedolce F Hatzichristodoulou G. Impact of resection technique on perioperative outcomes and surgical margins after partial nephrectomy for localized renal masses: a prospective multicenter study. J Urol. (2020) 203:496–504. 10.1097/JU.0000000000000591 31609167

[9] Minervini A Campi R Serni S Carini M. Re: Raj Satkunasivam, Sheaumei Tsai, Sumeet Syan, et al. Robotic unclamped “minimal-margin” partial nephrectomy: ongoing refinement of the anatomic zero-ischemia concept. Eur Urol. 2015; 68:705–12. Eur Urol. (2016) 70:e47–50. 10.1016/j.eururo.2015.12.037 26778463

[10] Minervini A Carini M Uzzo RG Campi R Smaldone MC Kutikov A. Standardized reporting of resection technique during nephron-sparing surgery: the surface-intermediate-base margin score. Eur Urol. (2014) 66:803–5. 10.1016/j.eururo.2014.06.002 24954792

[11] Bertolo R Campi R Mir MC Klatte T Kriegmair MC Salagierski M. Systematic review and pooled analysis of the impact of renorrhaphy techniques on renal functional outcome after partial nephrectomy. Eur Urol Oncol. (2019) 2:572–5. 10.1016/j.euo.2018.11.008 31412012

[12] Minervini A Campi R Di Maida F Mari A Montagnani I Tellini R. Tumor-parenchyma interface and long-term oncologic outcomes after robotic tumor enucleation for sporadic renal cell carcinoma. Urol Oncol. (2018) 36:527.e1–527.e11. 10.1016/j.urolonc.2018.08.014 30268711

[13] Delahunt B Cheville JC Martignoni G Humphrey PA Magi-Galluzzi C McKenney J. The International society of urological pathology (ISUP) grading system for renal cell car- cinoma and other prognostic parameters. Am J Surg Pathol. (2013) 37:1490. 10.1097/PAS.0b013e318299f0fb 24025520

[14] Srigley JR Delahunt B Eble JN Egevad L Epstein JI Grignon D. The International Society of Urological Pathology (ISUP) Vancouver classification of renal neo- plasia. Am J Surg Pathol. (2013) 37:1469. 10.1097/PAS.0b013e318299f2d1 24025519

[15] Alameddine M Koru-Sengul T Moore KJ Miao F Sávio LF Nahar B. Trends in Utilization of Robotic and Open Partial Nephrectomy for Management of cT1 Renal Masses. Eur Urol Focus. (2019) 5:482–7. 10.1016/j.euf.2017.12.006 29325761

[16] Cacciamani GE Okhunov Z Meneses AD Rodriguez-Socarras ME Rivas JG Porpiglia F. Impact of three-dimensional printing in urology: state of the art and future perspectives. A systematic review by ESUT-YAUWP Group. Eur Urol. (2019) 76:209–21. 10.1016/j.eururo.2019.04.044 31109814

[17] Veneziano D Amparore D Cacciamani G Porpiglia F. Climbing over the barriers of current imaging technology in urology. Eur Urol. (2020) 77:142–3. 10.1016/j.eururo.2019.09.016 31610902

[18] Antonelli A Cindolo L Sandri M Annino F Carini M Celia A. Predictors of the transition from off to on clamp approach during ongoing robotic partial nephrectomy: data from the CLOCK randomized clinical trial. J Urol. (2019) 202:62–8. 10.1097/JU.0000000000000194 30827166

[19] Hughes-Hallett A Pratt P Mayer E Martin S Darzi A Vale J. Image guidance for all TilePro display of 3-dimensionally reconstructed images in robotic partial nephrectomy. Urology. (2014) 84:237–42. 10.1016/j.urology.2014.02.051 24857271

[20] Wake N Rosenkrantz AB Huang R Park KU Wysock JS Taneja SS. Patient-specific 3D printed and augmented reality kidney and prostate cancer models: impact on patient education. 3D Print Med 5, 4 (2019). 10.1186/s41205-019-0041-3 30783869 PMC6743040

[21] Shakir A Fichtenbaum E Aron M. Robotic excision of tumor in isthmus of horseshoe kidney in the setting of prior endovascular AAA repair. Urol Video J. (2020) 8:100059. 10.1016/j.urolvj.2020.100059

[22] Bottero D Fanciullo C Bonomo G Ferro M de Cobelli O. Robotic-assisted laparoscopic simple enucleation in a horseshoe kidney. A case report and review of the literature. Urology. (2020) 143:5–10. 10.1016/j.urology.2020.03.034 32283171

[23] Grande P Campi R Rouprêt M. Relationship of surgeon/hospital volume with outcomes in uro-oncology surgery. Curr Opin Urol. (2018) 28:251–9. 10.1097/MOU.0000000000000490 29461273

[24] Mari A Campi R Schiavina R Amparore D Antonelli A Artibani W. Nomogram for predicting the likelihood of postoperative surgical complications in patients treated with partial nephrectomy: a prospective multicentre observational study (the RECORd 2 project). BJU Int. (2019) 124:93–102. 10.1111/bju.14680 30653796

[25] Antonelli A Mari A Longo N Novara G Porpiglia F Schiavina R. Role of clinical and surgical factors for the prediction of immediate, early and late functional results, and its relationship with cardiovascular outcome after partial nephrectomy: results from the prospective multicenter RECORd 1 Project. J Urol. (2018) 199:927–32. 10.1016/j.juro.2017.11.065 29154848

[26] Ficarra V Crestani A Bertolo R Antonelli A Longo N Minervini A. Tumour contact surface area as a predictor of postoperative complications and renal function in patients undergoing partial nephrectomy for renal tumours. BJU Int. (2019) 123:639–45. 10.1111/bju.14567 30253020

