# Supplementary figures and images for: Case Report: Optimizing Pre- and Intraoperative Planning With Hyperaccuracy Three-Dimensional Virtual Models for a Challenging Case of Robotic Partial Nephrectomy for Two Complex Renal Masses in a Horseshoe Kidney
Source: Front Surg. 2021 May 31;8:665328. doi: 10.3389/fsurg.2021.665328 (PMC8200488; doi:10.3389/fsurg.2021.665328)

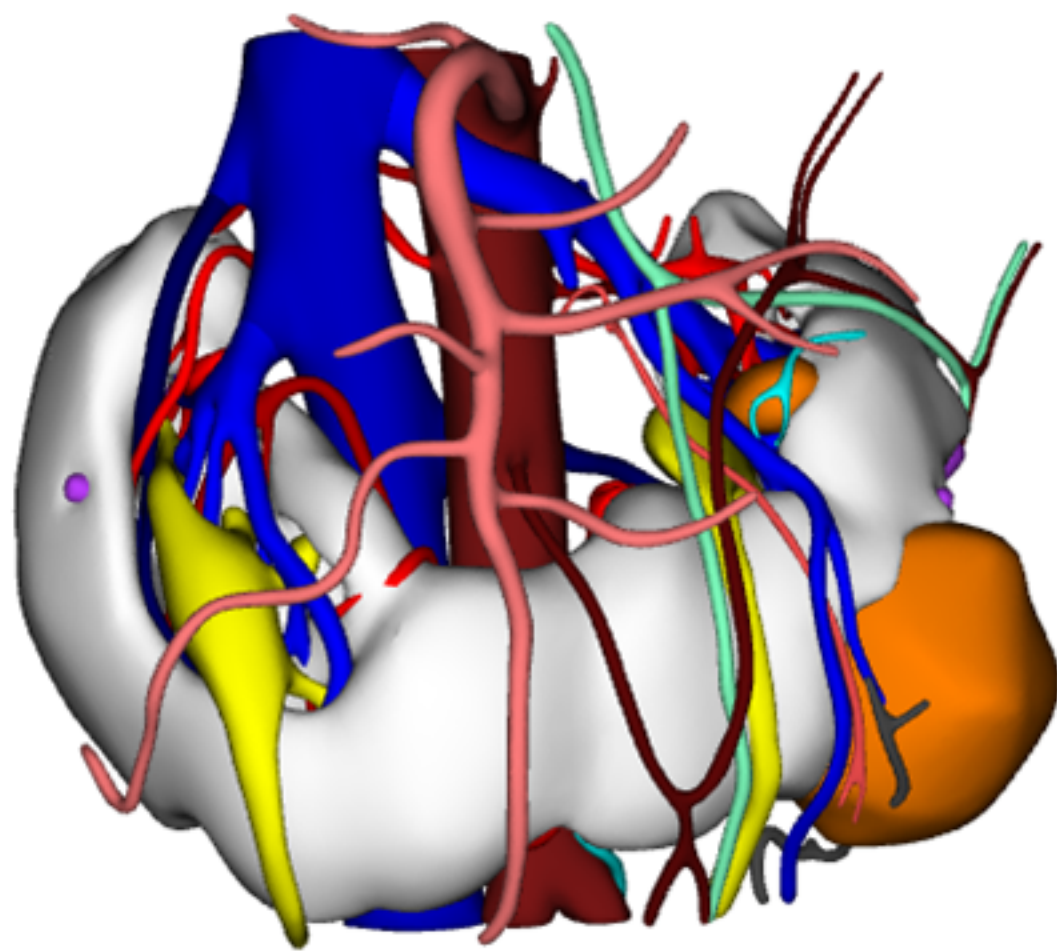

Supplement: Supplementary file 1 [file Data_Sheet_1.PDF]

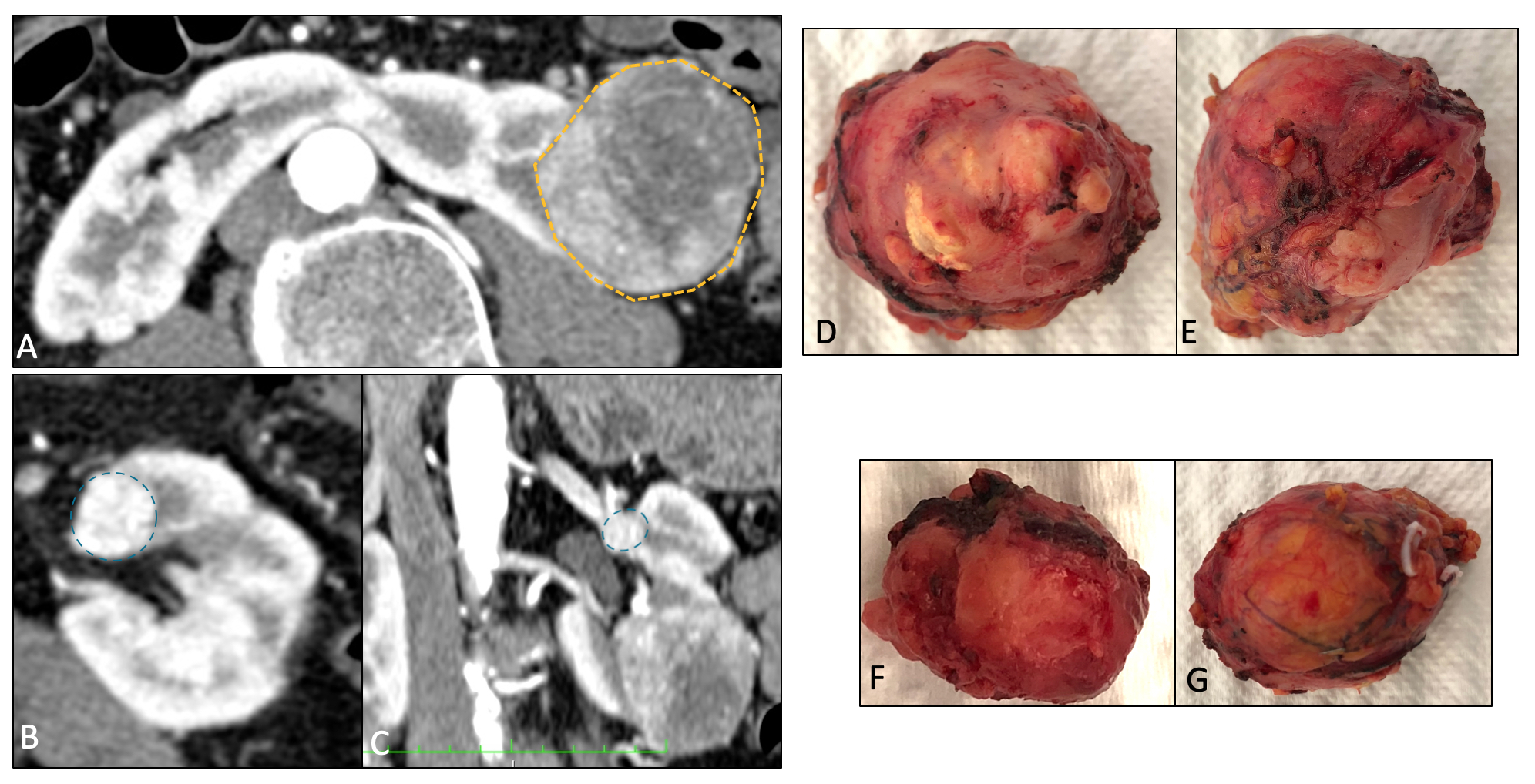

Supplement: Supplementary Figure 1 — Overview of the two renal masses resected in our case at both contrast-enhanced computed tomography (CT) scan (A-C) and histopathological analysis (D-G). The larger renal mass was revealed to be a 52 x 50 mm, G3, clear cell renal cell carcinoma (ccRCC) with evidence of necrosis and negative surgical margins (pT1bNxMx) (despite the presence of a small capsulotomy – see Figure 4) while the smaller mass a 20 x 13 mm, G3 ccRCC without necrosis with a focal involvement of the perirenal fat and negative surgical margins (pT3aNxMx). [file Image_1.JPEG]
