# Supplementary material for: Case Report: Optimizing Pre- and Intraoperative Planning With Hyperaccuracy Three-Dimensional Virtual Models for a Challenging Case of Robotic Partial Nephrectomy for Two Complex Renal Masses in a Horseshoe Kidney
Source: Front Surg. 2021 May 31;8:665328. doi: 10.3389/fsurg.2021.665328 (PMC8200488; doi:10.3389/fsurg.2021.665328)
Supplement: Supplementary file 2 [file Data_Sheet_2.docx]

**List of Supplementary Videos**

**Figure 1.** https://vimeo.com/manage/videos/508768220

**Figure 2.** https://vimeo.com/manage/videos/508753750

**Figure 4.** https://vimeo.com/manage/videos/508826651
